# The future of medical debt relief in the United States

**DOI:** 10.1093/haschl/qxag286

**Published:** 2026-10-15

**Authors:** Alex J Dong, Victor C Agbafe, Nicholas L Berlin

**Affiliations:** Harvard Medical School, Boston, MA 02115, United States; Harvard Business School, Boston, MA 02115, United States; University of Michigan Medical School, Ann Arbor, MI 48109, United States; Harvard Medical School, Boston, MA 02115, United States; Department of Surgery, Brigham and Women's Hospital, Boston, MA 02115, United States

**Keywords:** health care finance, health care affordability, health care costs, medical debt, debt relief, access to care, socioeconomic factors, health equity, health disparities, state policy, Medicaid, government programs, health policy

## Abstract

Over 100 million Americans carry an estimated $220 billion in medical debt, contributing to two-thirds of personal bankruptcies. Since 2022, state and local governments have responded by purchasing and canceling residents’ medical debt. In this scoping review, we examine and compare the structure and impact of these medical debt relief efforts. In sum, 10 states and 21 local governments have eliminated $13.5 billion in medical debt for 7 million residents to date. However, we found that these efforts have been primarily funded by temporary federal pandemic relief dollars that expire after 2026. North Carolina is a notable exception: by conditioning enhanced hospital Medicaid payments on the adoption of debt-mitigation policies, the state has erased $6.5 billion in medical debt among its residents, representing 48% of all relief nationally, without utilizing federal pandemic funds or general state-fund appropriations. We argue that leveraging payment incentives may potentially offer a more durable approach to relieving existing debt and that pairing retrospective debt relief with structural consumer protections is vital to target the upstream drivers of future debt burden. We also argue that sustaining progress requires evaluating program costs and the understudied health and financial outcomes of beneficiaries.

Key points

- From 2022 to 2026, 10 states and 21 local governments have eliminated a combined $13.5 billion in medical debt for 7 million Americans, but nearly all of these efforts rely on temporary federal pandemic funds that expire at the end of 2026.
- States may create more sustainable debt relief efforts by linking these programs to hospital payment incentives: North Carolina's model that conditioned enhanced Medicaid payments on hospitals’ adoption of debt-mitigation policies eliminated $6.5 billion of debt representing 48% of all national relief, without using temporary pandemic funds.
- Pairing debt relief efforts with structural consumer protections and evaluating the understudied health and financial outcomes of beneficiaries is vital in targeting both the symptoms and upstream causes of the medical debt crisis.

Medical debt in the United States has reached a crisis point. Over 100 million Americans carry an estimated $220 billion in medical debt, a quarter of whom owe more than $5000.^1^ Around two-thirds (66.5%) of all personal bankruptcies are attributable to medical debt, either through high costs or lost income.^2^

Recently, state and local debt relief programs have begun to provide relief. In July 2022, Cook County, Illinois, became the first US jurisdiction to partner with the nonprofit Undue Medical Debt, using American Rescue Plan Act (ARPA) funds, a $1.9 trillion federal post-pandemic stimulus law, to purchase and cancel residents’ medical debt.^3^ Thirty state and local governments have followed suit.^3^ However, with the expiration of ARPA funds after 2026, the future of these initiatives is uncertain.

Here, we compare state and local debt relief initiatives and offer policy recommendations to build a more sustainable path forward for both retrospective debt relief and future debt prevention.

## Current state and local debt relief programs

We conducted a scoping review of state- and local-level medical debt relief programs between January 2022 and April 2026. Ten states (NC, NJ, IL, AZ, CT, MI, DE, RI, PA, VT) and 21 local governments have eliminated a combined $13.5 billion in medical debt for 7 million residents since 2022 (Figure 1). All states, except for North Carolina, have established “buy-and-abolish” models using a combination of ARPA and general appropriations to purchase bundled debt, with an investment-to-debt-relief ratio of approximately $1 to $100.

**Figure 1.**
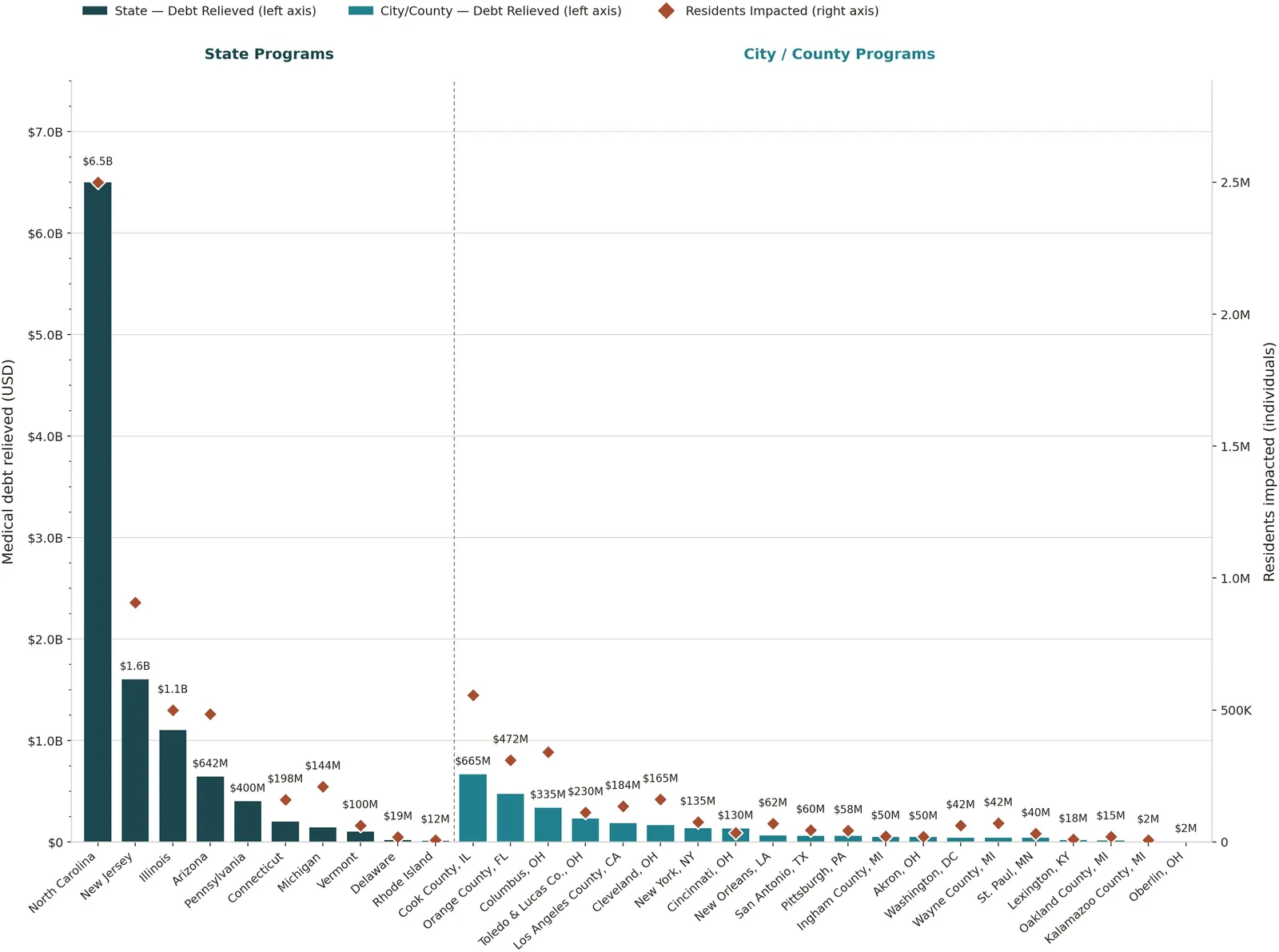
Medical debt relieved and residents impacted, by US Jurisdiction (2022-2026). Programs were identified through Undue Medical Debt's published government partnership list, the Budget Equity Project from the Institute of Race, Power and Political Economy, and publicly available sources such as gubernatorial press releases, state agency announcements, and Kaiser Family Foundation (KFF) analyses. For each jurisdiction with active debt relief efforts, we compiled program characteristics—launch year, eligibility criteria, and funding sources (American Rescue Plan Act [ARPA], general fund, or Medicaid state-directed payment)—as well as reported outcomes through April 30, 2026 (debt eliminated and number of beneficiaries). From 2022 to 2026, 10 states and 21 local governments have eliminated a combined $13.5 billion in medical debt for 7 million residents across the United States. The left Y-axis reports medical debt relieved in US dollars; the right Y-axis reports residents impacted. Note: Pennsylvania and Oberlin, OH do not report residents impacted and therefore have no diamond marker.

Those whose household income is ≤400% of the federal poverty level (FPL) or who carry medical debt comprising at least 5% of household income typically qualify for relief under Undue Medical Debt's standard hardship thresholds.^3^ Some debt relief programs also have minimum debt age eligibility thresholds, like Vermont's at >18 months. The programs differ in their consideration of dental expenditures, ambulance services, and behavioral health costs.

In contrast to buy-and-abolish models, North Carolina has offered hospitals enhanced Medicaid reimbursement contingent on participation in comprehensive debt relief efforts. All 99 North Carolina acute-care hospitals opted into the program, eliminating $6.5 billion in debt for 2.5 million North Carolina residents and accounting for 48% of total relief across the country without using ARPA or state funds.^4^ North Carolina's program also included several policies intended to reduce the creation of future debt: all participating hospitals agreed to erase Medicaid enrollees’ medical debt since 2014; auto-enroll patients eligible for Medicaid, food assistance programs, or homeless charity care programs; and provide discounts (50%-100%) on existing debt for patients at ≤300% FPL.

## Approaches to the sustainable financing of medical debt relief

With ARPA funds expiring by the end of 2026, the buy-and-abolish model is unsustainable, despite recent philanthropy. Programs that leverage payment incentives may offer improved long-term viability. North Carolina's approach, which conditions enhanced Medicaid payments on hospitals’ adoption of debt-mitigation policies, offers lessons for other states with Medicaid managed-care arrangements, although its transferability depends on financing capacity, federal approval, and new payment limits.

North Carolina's incentive structure is based on the Healthcare Access and Stabilization Program (HASP), a state-directed payment requiring managed-care plans to pay hospitals up to average commercial rates. North Carolina finances enhanced Medicaid hospital payments through hospital assessments covering the nonfederal share and federal matching funds covering the remainder, increasing Medicaid spending but using neither ARPA funds nor state general-fund appropriations.^4^ Authorized by state legislation alongside Medicaid expansion in 2023, HASP's payment arrangement requires annually renewed Centers for Medicare and Medicaid Services (CMS) approval and relies on public funding, hospital participation, and state oversight. However, the 2025 federal budget reconciliation law limits new state-directed payments near Medicare rates while phasing down existing ones starting in 2028, constraining North Carolina's long-term financing and leaving other states with smaller incentives.^5^

Despite these changes, the success of North Carolina's approach suggests that states may create more sustainable programs by seeking opportunities to link hospital payment incentives with commitments to cancel and prevent medical debt. Of note, states that have not expanded Medicaid may be unable to condition Medicaid payment enhancements on hospital participation in the first place, which may hinder debt relief efforts for populations in greatest need, as medical debt is often higher and disproportionately impacts low-income individuals in such states.^6^

## Reducing the incurrence of future medical debt

Debt relief programs often treat the symptoms, rather than the root causes, of unaffordable healthcare. Pairing debt relief with structural consumer protections targets the medical debt crisis one step upstream, an approach that four states (NC, NJ, DE, VT) and Los Angeles County have already adopted. In New Jersey, the Louisa Carman Medical Debt Relief Act prohibits creditors from reporting medical debt to credit bureaus, caps interest rates at 3%, and prohibits wage garnishment for incomes <600% FPL. Oregon enacted House Bill 3320, which mandates patient screening for financial assistance before being billed for care, caps interest rates, and prohibits predatory debt collection practices such as wage garnishment and property liens.

## Debt relief program evaluation and outcomes

Empirical data on the downstream health and financial impacts of debt relief remain scarce, as state and local governments typically report only the amount of debt abolished and recipients impacted, not beneficiary outcomes. One empirical study investigating the downstream effects of debt-cancellation interventions showed modest health and financial impacts at best, which may be at odds with the expectations of proponents of medical debt relief and self-reported assessments of debt relief recipients.^7^ Outcomes of debt relief that remain important to evaluate include healthcare utilization, mental and physical health outcomes, credit outcomes, debt accumulation, and financial well-being. A separate study of the impact of prospective financial assistance programs found that they improved care utilization and chronic disease management.^8^ Given that costs can present barriers to obtaining care in the first place, upstream programs should be evaluated primarily on the basis of their impact on healthcare access and utilization, as well as associated health outcomes. State and local governments can partner with foundations to collect beneficiary data on these long-term outcomes for the evaluation of both upstream and downstream interventions. Moreover, a coordinated multi-state infrastructure can link credit bureau claims and survey data to investigate the impact of debt relief efforts on consumer spending and local economies.

## Looking ahead

Ultimately, addressing the medical debt crisis in the United States requires action at multiple levels. To sustain medical debt relief efforts after ARPA, states should secure ongoing financing for targeted relief, seek new strategies to link hospital payment incentives with commitments to cancel existing debt, and pair these efforts with structural consumer protections that reduce future debt. Where financing capacity and federal approval permit, enhanced Medicaid payments could be conditioned on debt cancellation, automatic financial assistance, and limits on harmful collection practices, like in North Carolina's approach. Where payment incentives are less feasible, states may pursue financial assistance and collection standards directly. North Carolina's model offers one approach to aligning hospital payment incentives with patients’ financial protection, although its durability and transferability remain uncertain. Evaluating program costs and beneficiaries’ health and financial outcomes will be vital to guiding future medical debt relief efforts. As temporary funding expires, sustained policy action is needed to build on the relief already provided, decrease future debt burden, and help shoulder the costs of care that too often fall on patients alone.

## Supplementary Material

qxag286_Supplementary_Data
